# Is there still need for microelectrode recording now the subthalamic nucleus can be well visualized with high field and ultrahigh MR imaging?

**DOI:** 10.3389/fnint.2015.00046

**Published:** 2015-08-11

**Authors:** Ersoy Kocabicak, Onur Alptekin, Linda Ackermans, Pieter Kubben, Mark Kuijf, Erkan Kurt, Rianne Esselink, Yasin Temel

**Affiliations:** ^1^Department of Neurosurgery, Maastricht Medical CenterMaastricht, Netherlands; ^2^Department of Neuroscience, Maastricht University Medical CenterMaastricht, Netherlands; ^3^Department of Neurosurgery, Ondokuz Mayis UniversitySamsun, Turkey; ^4^Department of Neurology, Maastricht Medical CenterMaastricht, Netherlands; ^5^Department of Neurosurgery, Donders Institute for Cognition, Brain and Behaviour, Radboud University Medical CenterNijmegen, Netherlands; ^6^Department of Neurology, Donders Institute for Cognition, Brain and Behaviour, Radboud University Medical CenterNijmegen, Netherlands

**Keywords:** microelectrode recording, magnetic resonance imaging, subthalamic nucleus, deep brain stimulation, Parkinson's disease

## The question

High frequency stimulation of the subthalamic nucleus (STN) is an effective treatment for patients with Parkinson's disease (PD) (Odekerken et al., 2012; Kocabicak et al., 2013; Schuepbach et al., 2013). The technique has been further refined throughout the years by improved magnetic resonance imaging (MRI) techniques, advanced neurophysiological recording possibilities, and advances in hardware and software technology (Kocabicak and Temel, 2013). There are at least two major determining factors for an acceptable therapeutic outcome: patient selection (Deuschl et al., 2006) and the accuracy of targeting of the relatively small STN (Temel et al., 2005). The latter requires a state-of-the art stereotactic approach, adequate imaging facilities, and a detailed neurophysiological mapping of the target area. The preferred area within the STN is the motor part (thought to be located dorsolaterally in the STN), which can, be to some extent, identified by intraoperative multi-unit activity analyses, and MRI-based tractography (Zaidel et al., 2010; Brunenberg et al., 2011).

While the STN could not be visualized on MRI images when modern DBS of the STN surgeries started in Grenoble in 1993, nowadays its visualization has become a routine procedure for most centers offering DBS for patients with PD. While using intraoperative electrophysiology was evident in the beginning, now it is questioned whether it still has an essential added value. In this opinion article, we aim to provide an answer on the question whether or not electrophysiology still has a clinically relevant role in this era of advanced neuroimaging technology, which enables us to visualize both function and structure anatomy.

## Old debate

The discussion of whether or not to use intraoperative microelectrode recording (MER) is not a new one (Hariz, 2002). This discussion was perhaps less vivid when modern DBS started to be applied in patients with PD. The STN was an invisible target on MR images in most centers and MER was considered very helpful to find and delineate the boundaries of the target (Pollak et al., 1993; Limousin et al., 1995; Shamir et al., 2012). Since then things have changed. However, currently the STN can be directly visualized on T2 weighed and susceptibility weighed MR images. The imaging field progresses rapidly further with ultra-high field imaging modalities becoming now available for patients (Plantinga et al., 2014).

It is more than 15 years ago that that the visualization of the STN for DBS surgeries was described (Starr et al., 1999). Mostly, T2 weighed and inversion recovery MRI sequences have been used. In most of the patients, the predefined target on T2 weighed MR images was chosen for implantation after intra-operative electrophysiology and test-stimulation (Bejjani et al., 2000; Egidi et al., 2002; Starr et al., 2002). This meant that in most patients MRI images could reliably show the STN, except for the y axis, in which microelectrode recording (MER) indicated that the STN extended more anteriorly than suggested by MRI (Hamani et al., 2005). Detailed volumetric analysis of MER-determined borders of the STN and MRI- defined borders in 22 patients (44 STN's), showed that MER-determined borders of the STN were exceeding the MRI signal (Schlaier et al., 2011). In addition, we examined the entry and exit borders of the STN on MRI images and with MER, using the probe's eye trajectory (Kocabicak et al., 2013). We found that T2 weighed MRI could reliably predict the electrophysiological entry and exit of the STN. Although these data confirm the accuracy of MRI in visualizing the STN, there are also limitations. There are known variations between the patients with respect to the x, y, and z planes, and the borders can sometimes be less clear, mainly toward the substantia nigra pars reticulata (SNr) (Hamani et al., 2005; Kocabicak et al., 2013).

## From atlas-based to MRI based coordinates and from single-electrode to multiple-electrode recordings

In our previous series of 55 patients with PD who underwent DBS of the STN, atlas- based coordinates were used and in about one third of the patients the predefined target (central trajectory) was used for final electrode implantation, after MER and intra-operative test-stimulation (Temel et al., 2007). With applying individually adjusted coordinates based on T2 weighed MRI, the central trajectory was chosen in about two-thirds of the patients (Kocabicak et al., 2013; Tonge et al., in press). This has resulted in a clear reduction in operation time. Similar rates have been reported by others with atlas-based (Amirnovin et al., 2006) and MRI-based targeting coordinates (Reck et al., 2012). The change from 1.5 to 3.0 T has also improved the accuracy of targeting (Toda et al., 2009; Kerl et al., 2012).

Another development has been the change of single-electrode to multiple-electrode intra-operative electrophysiological recordings (Temel et al., 2007). The latter provides more detailed information about the electrophysiological boundaries of the STN; however, implantation of several electrodes at one time might increase the risk of bleeding. We found that the simultaneous implantation of multiple electrodes did not cause more bleedings or other major intracranial complication. The use of multiple electrodes resulted in better motor results when compared with patients who underwent DBS of the STN guided with a single recording electrode. There are reports, however, suggesting increased risk of hemorrhage due to MER (Ben-Haim et al., 2009; Xiaowu et al., 2010).

## Back to the question

Is intra-operative electrophysiology necessary to find the STN? Our answer is no based on the advances in MRI technology.

In line with this experienced DBS centers have shown good outcome with a MRI-guided approach (Ostrem et al., 2013; Aviles-Olmos et al., 2014). So should we abandon MER then? In our centers, we have decided not to abandon it for a number of reasons. Even in experienced centers, in about two-thirds of the cases, the predefined target is chosen for final implantation. In one-third, an alternative trajectory is needed. With MER, alternative trajectories are immediately available. The trajectory with the second longest and, if needed, the third longest STN activity can be used as alternative trajectories. Two other less common reasons to use intra-operative electrophysiology can be an unexpected error in the stereotactic approach or a shift caused by excessive CSF leakage or a hematoma (Reck et al., 2012).

### Conflict of interest statement

The Review Editor Dr. Hagai Bergman declares that, despite chairing meetings for the Mediterranean Neuroscience Society as well as organizing an academic course with Author Dr. Yasin Temel (as a joint collaboration between Maastricht University Medical Center and the Mediterranean Neuroscience Society) the review process was handled objectively. The authors declare that the research was conducted in the absence of any commercial or financial relationships that could be construed as a potential conflict of interest.
